# Oncolytic viruses and checkpoint inhibitors: combination therapy in clinical trials

**DOI:** 10.1186/s40169-018-0214-5

**Published:** 2018-11-14

**Authors:** Christopher J. LaRocca, Susanne G. Warner

**Affiliations:** Division of Surgical Oncology, Department of Surgery, City of Hope National Medical Center, 1500 E Duarte Road, Duarte, CA 91010 USA

**Keywords:** Immune checkpoint inhibitors, Oncolytic viral therapy, Clinical trials, Immunotherapy, Combination therapy

## Abstract

Advances in the understanding of cancer immunotherapy and the development of multiple checkpoint inhibitors have dramatically changed the current landscape of cancer treatment. Recent large-scale phase III trials (e.g. PHOCUS, OPTiM) are establishing use of oncolytic viruses as another tool in the cancer therapeutics armamentarium. These viruses do not simply lyse cells to achieve their cancer-killing effects, but also cause dramatic changes in the tumor immune microenvironment. This review will highlight the major vector platforms that are currently in development (including adenoviruses, reoviruses, vaccinia viruses, herpesviruses, and coxsackieviruses) and how they are combined with checkpoint inhibitors. These vectors employ a variety of engineered capsid modifications to enhance infectivity, genome deletions or promoter elements to confer selective replication, and encode a variety of transgenes to enhance anti-tumor or immunogenic effects. Pre-clinical and clinical data have shown that oncolytic vectors can induce anti-tumor immunity and markedly increase immune cell infiltration (including cytotoxic CD8^+^ T cells) into the local tumor microenvironment. This “priming” by the viral infection can change a ‘cold’ tumor microenvironment into a ‘hot’ one with the influx of a multitude of immune cells and cytokines. This alteration sets the stage for subsequent checkpoint inhibitor delivery, as they are most effective in an environment with a large lymphocytic infiltrate. There are multiple ongoing clinical trials that are currently combining oncolytic viruses with checkpoint inhibitors (e.g. CAPTIVE, CAPRA, and Masterkey-265), and the initial results are encouraging. It is clear that oncolytic viruses and checkpoint inhibitors will continue to evolve together as a combination therapy for multiple types of cancers.

## Background

Immunotherapy is at the forefront of cancer research and treatment with the American Society of Clinical Oncology (ASCO) naming immunotherapy as the advance of the year in both 2016 and 2017 [1, 2] and specifically citing adoptive cell immunotherapy as this year’s most important advancement [3]. The large number of clinical trials currently employing immunotherapeutic agents is a testament to the monumental advances they are making in cancer treatment.

Individual immunotherapies have demonstrated remarkable treatment effects in melanoma, lung cancer, and multiple intra-abdominal malignancies [4]. In particular, a class of drugs known as checkpoint inhibitors has been of great interest to researchers and clinicians (Table 1). These antibodies block the negative regulators of T cell function (immune checkpoints), thereby increasing T-cell activation [4, 5]. The United States Food and Drug Administration (FDA) first approved ipilimumab (a monoclonal antibody inhibiting cytotoxic T lymphocyte-associated antigen-4 [CTLA-4]) for the treatment of metastatic melanoma in 2011 [5]. A few years later in 2014, the FDA approved nivolumab and pembrolizumab (both monoclonal antibodies targeting programmed death receptor 1 [PD-1]) for the treatment of advanced melanoma [6].

**Table 1:** Currently approved checkpoint inhibitors

Drug name	Target	Manufacturer	Approved disease site
Ipilimumab	CTLA-4	Bristol-Meyers Squibb	Melanoma RCC^a^
Pembrolizumab	PD-1	Merck	Melanoma NSCLC HNSCC HL PMBCL Urothelial carcinoma MSI-H/dMMR Solid Tumors Gastric Cancer Cervical Cancer
Nivolumab	PD-1	Bristol-Meyers Squibb	Melanoma RCC NSCLC HNSCC HL Urothelial carcinoma Colorectal cancer HCC
Avelumab	PD-L1	Merck/Pfizer	Merkel cell carcinoma RCC* Urothelial carcinoma
Durvalumab	PD-L1	Astra Zeneca	Urothelial carcinoma NSCLC
Atezolizumab	PD-L1	Genentech	Urothelial carcinoma NSCLC

*RCC* renal cell cancer, *NSCLC* non-small cell lung cancer, *HNSCC* head and neck squamous cell carcinoma, *HL* Hodgkin lymphoma (classic), *PMBCL* primary mediastinal B cell lymphoma, *MSI-H* microsatellite instability high, *dMMR* mismatch repair gene deficient, *HCC* hepatocellular carcinoma

^a^Approval granted for use in combination with another therapeutic agent

The principle of combining therapeutics with complementary mechanisms has also been applied to checkpoint inhibition across a range of malignancies including gastrointestinal and soft tissue cancers [4, 7]. For instance, the Checkmate 067 trial demonstrated the effect of the combination of ipilimumab and nivolumab in patients with untreated melanoma [8]. In these patients with advanced disease, the combination of these two agents resulted in a vast overall survival improvement at 3 years compared with ipilimumab alone (58% vs 34%) [8, 9].

The rapidly advancing field of clinical oncolytic virotherapy is itself coming to be understood as a unique type of immunotherapy. Oncolytic viruses are naturally occurring or genetically modified viruses that infect, replicate in, and kill cancer cells without harming normal cells [10]. Recent decades have seen dramatic advances in gene manipulation capabilities and thus improvements in vector design [11]. Additionally, the understanding of how an oncolytic adenovirus alters the local tumor microenvironment (TME) has led some to think of the field as ‘oncolytic immunotherapy’. Following viral infection, there are increased levels of local cytokine expression as well as an influx of immune cells including natural killer (NK) cells, activated T cells, and antigen presenting cells (APC) [12]. Furthermore, PD-L1 expression is known to increase on tumor and immune cells following viral infection [13]. Taken together, these changes alter the local TME and change it from ‘cold’ to ‘hot’ with a flood of cytokines and immune effectors.

Checkpoint inhibition works best when there is a large lymphocytic infiltrate, which is not always the case for a given tumor [13, 14]. The changes to the local TME following oncolytic virus delivery creates a situation that can be exploited with novel combination regimens, namely oncolytic vectors and checkpoint inhibitors. The efficacy of combining oncolytic viruses and checkpoint inhibition has been shown in pre-clinical data, and there are currently more than 15 ongoing clinical trials employing a combination regimen of these two types of cancer therapeutics (Table 2). With the abundance of ongoing pre-clinical and clinical studies, it is certain that the futures of viral oncolysis and checkpoint inhibition will be intertwined.

**Table 2:** Important ongoing clinical trials combining oncolytic vectors and checkpoint inhibitors

Trial identifier	Study phase	Virus type	Virus name	Virus dose, schedule	Virus route	Checkpoint inhibitor	Study regimen	cancer type
NCT 03004183 STOMP	II	Adenovirus	ADV/HSV-tk	5x10^11^ vp, single injection	IT	Pembrolizumab	Virus (Day 0), Valacyclovir (Day1–15), SBRT (Day 2–16, total 30 Gy), CI (starting day 22)	Metastatic NSCLC Metastatic TNBC
NCT 02798406 CAPTIVE/KEYNOTE-192	II	Adenovirus	DNX-2401	5x10^8^–5x10^10^ vp, single injection	IT	Pembrolizumab	Virus (Day 0), CI (starting Day 7–9)	Glioblastoma, gliosarcoma
NCT 03003676	I	Adenovirus	ONCOS-102	3 × 10^11^ vp, multiple injection (× 3)	IT	Pembrolizumab	Cyclophosphadmide priming, Virus (Day 1, 4, 8), CI (starting day 22)	Advanced/Unresectable Melanoma Progressing After PD1 Blockade
NCT 03408587 VLA-024 CLEVER	Ib	Coxsackie	CAVATAK (CVA21)	1 × 10^9^ TCID50, multiple doses	IV	Ipilimumab	Virus (Day 1, 3, 5, 8 then repeat cycle every 21 days for up to 8 cycles) + CI (Day 8, 29, 50, 71)	Uveal Melanoma with Liver Metastases
NCT 02565992 VLA-011 CAPRA	I	Coxsackie	CAVATAK (CVA21)	4.5 × 10^6^ TCID50/kg, multiple injections	IT	Pembrolizumab	Virus (Day 1, 3, 5, 8, then 3 week intervals), CI (starting day 8)	Advanced Melanoma
NCT 02824965	I, II	Coxsackie	CAVATAK (CVA21)	1 × 10^8^–1 × 10^9^ TCID50, multiple injections	IT	Pembrolizumab	Virus (Day 1, 3, 5, 8, 29, 50, 71, 92, 113, 134, 155) + CI (starting day 8)	Advanced NSCLC
NCT 03153085	II	HSV	HF10 (TBI-1401)	1 × 10^7^ TCID50/mL, multiple injections (× 6)	IT	Ipilimumab	Virus (Week 1, 2, 3, 4, 7, 10) + CI (3-week intervals × 4 doses)	Unresectable/Metastatic Melanoma in Japanese Patients
NCT 02272855	II	HSV	HF10 (TBI-1401)	1 × 10^7^ TCID50/mL, multiple injections (× 6)	IT	Ipilimumab	Virus (Week 1, 2, 3, 4, 7, 10) + CI (3-week intervals × 4 doses)	Unresectable/Metastatic Melanoma
NCT 03259425	II	HSV	HF10 (TBI-1401)	1 × 10^7^ TCID50/mL, multiple injections	IT	Nivolumab	Virus (Day 0, 7, 14, 21, 28, 42, 56, 70, 84) + CI (starting day 0, every 2 weeks for 7 doses)	Resectable Stage IIIB/C, IV Melanoma
NCT 01740297	Ib, II	HSV	TVEC (Talimogene Laherparepvec)	10^6^ PFU/mL, multiple injections	IT	Ipilimumab	Virus (Week 1, 4, then every 2 weeks) + CI (Week 1, then every 3 weeks for 4 total doses)	Unresected Stage IIIb/IV melanoma
NCT 02263508 Masterkey 265/KEYNOTE-034	Ib, III	HSV	TVEC (Talimogene Laherparepvec)	Multiple injections	IT	Pembrolizumab	Virus (Day 1, then every 2–3 weeks) + CI (starting 2–5 weeks after first viral inoculation)	Unresectable Stage IIIb/IV Melanoma
NCT 02626000 Masterkey 232/Keynote-137	Ib, III	HSV	TVEC (Talimogene Laherparepvec)	10^6^ PFU/mL, multiple injections	IT	Pembrolizumab	Virus (Day 1 and every 3 weeks) + CI (Day 1 and every 3 weeks)	Recurrent/Metastatic HNSCC
NCT 02879760	I, II	Maraba Virus	MG1-MAGEA3	1x10^10^–3x10^11^ pfu, multiple doses	IV	Pembrolizumab	Ad/MAGEAE priming, MG1-MAGEA3 (Day 15/18), CI starting day 22	Previously treated NSCLC
NCT 02620423	Ib	Reovirus	Reolysin (Pelareorep)	4.5 × 10^10 ^TCID50, multiple doses	IV	Pembrolizumab	Virus (Day 1, 2), Chemo: Gemcitabine or Irinotecan or 5-FU/LV (Day 1), CI (starting Day 8)	Pancreatic Adenocarcinoma
NCT 03206073	I, II	Vaccinia	Pexa Vec (Pexastimogene Devacirepvec)	3 × 10^8^–1 × 10^9^ pfu, multiple doses (× 4)	IV	Durvalumab Tremelimumab	Virus (Day 1, 2, 16 of cycle 1; Day 2 of cycle 2) + CI (Day 1 of each cycle)	Refractory Colorectal Cancer
NCT 02977156 ISI-JX	I	Vaccinia	Pexa Vec (Pexastimogene Devacirepvec)	1 × 10^9^ pfu, multiple injections	IT	Ipilimumab	Virus (Week 1, 3, 5, 9, 12) + CI (Week 3, 5, 9, 12–IT injection)	Metastatic/Advanced Solid Tumors
NCT 03071094	I, IIa	Vaccinia	Pexa Vec (Pexastimogene Devacirepvec)	1 x 10^9^ pfu, multiple injections	IT	Nivolumab	Virus (Day 1, Day 14, Day 28) + CI (starting day 14)	Advanced HCC

*VP* viral particle, *pfu* plaque forming unit, *TCID* tissue culture infective dose, *IT* intratumoral, *IV* intravenous, *SBRT* stereotactic body radiotherapy, *CI* checkpoint inhibitor, *NSCLC* non small cell lung cancer, *TNBC* triple negative breast cancer, *HNSCC* head and neck squamous cell carcinoma, *HCC* hepatocellular carcinoma

In this review, we will explore the combination of oncolytic virotherapy with checkpoint inhibitors. There are many different vector platforms under investigation, all of which are in different stages of development. We will look at a sampling of pre-clinical data, published human trials, and highlight important ongoing clinical trials.

## Oncolytic viruses

Current-generation oncolytic viral vectors can be engineered to target specific types of cancer cells, selectively replicate within them, and locally express a transgene [11]. The tumor tropism of a virus is a key property that is essential to maximize cancer-killing effects on the tumor, while minimizing the damage to surrounding normal tissues. One approach to optimizing vector replication selectivity is via a tumor-specific or tissue-specific promoter element that is incorporated into the viral genome. Prostate-specific antigen (PSA), cyclooxygenase-2 (Cox2), and human telomerase reverse transcriptase (TERT) promoters are just a few examples of promoter sequences that can be used to confer selective viral replication to target tissues [15–19]. Additionally, deletions in key portions of the viral genome can also allow for selective viral replication. For example, deletions in the E1 region of the adenoviral genome can cause oncolytic adenoviruses to not replicate in normal cells due to the absence of key viral protein products [20]. Then, there must be additional pathway or signaling alterations to facilitate oncolytic virus replication in tumors. One example in many cancers is a defect in the Retinoblastoma (RB)-E2F pathway, which ultimately allows a virus to replicate as its normally negative regulatory function is absent [16]. Also, regulatory proteins from certain cancers (such as the human papilloma virus E6 and E7 oncoproteins) can functionally transcomplement missing viral proteins (such those in an adenovirus with E1 deletions) to allow for viral replication [21, 22]. No matter the mechanism, a key component of any well-designed vector is the ability to selectively replicate in target cells of interest.

As gene therapy approaches have improved, researchers have been able to insert numerous transgenes into multiple different vector platforms to achieve a variety of effects. The size of the viral genome affects the transgene capacity, which makes certain vector systems with larger genomes more desirable. Genes encoding interferon alpha, granulocyte macrophage colony stimulating factor (GM-CSF), and multiple cytokines have all been used as transgenes in oncolytic vectors [12, 23–25]. In addition, the sodium iodide symporter (NIS) gene has been used to allow for monitoring of viral replication [26, 27]. The ability to tailor transgene-insertion to the unique purposes of an individual vector makes oncolytic virotherapy a versatile tool in the cancer treatment armamentarium.

Following viral inoculation (especially through an intravenous route), circulating antibodies and the complement cascade can negate the efficacy of an oncolytic virus [11]. As many patients have previously encountered viruses due to vaccination or environmental exposure, it is no surprise that there is a high incidence of neutralizing antibodies to some oncolytic viruses [16]. These effects are often more pronounced following the second and subsequent doses of virus which may serve as a boost to the existing immune response; consequently, researchers have developed multiple approaches to minimize virus neutralization [28]. Strategies include using alternate serotypes, shielding the virus by PEGylation of the viral coat or polymer coating, suppression of the host immune system, and using a carrier (such as mesenchymal stem cells) to deliver the virus to the tumor bed [11, 16, 29–31].

Improved infection efficiency, highly-selective replication, and transgene expression make modern-day oncolytic viruses a robust cancer therapeutic that are readily adaptable to combination therapy with other anti-cancer agents.

## The immune response and checkpoint inhibitors

The lytic effects of an oncolytic virus only represent a portion of virally-induced cancer-killing potential. Another main component of viral-mediated killing results from the vector’s interactions with the immune system. Immunogenic cell death (ICD) can occur through multiple different mechanisms including autophagy, necrosis, and apoptosis with each of these inducing a different degree and type of immune response [32], but none of these are adequate to fully characterize the complex interactions that result in oncolytic virus-mediated cell death [11].

Virus-induced oncolysis can cause the dying cancer cell to release damage associated molecular patterns (DAMPs). These entities (which include cell surface proteins, membrane proteins, and nucleic acids) are released following cell death and can serve as ‘danger signals’ to prime the immune system [33]. Pathogen-associated molecular patterns (PAMP) are produced by various types of microorganisms (including viruses) and are ultimately recognized by pattern recognition receptors (PRRs) in the innate immune system [34]. These ‘danger signals’ are then picked up by antigen presenting cells (APC) such as dendritic cells (DC) and presented to T cells, which then can potentially initiate a systemic, adaptive immune response [12].

**Fig. 1:**
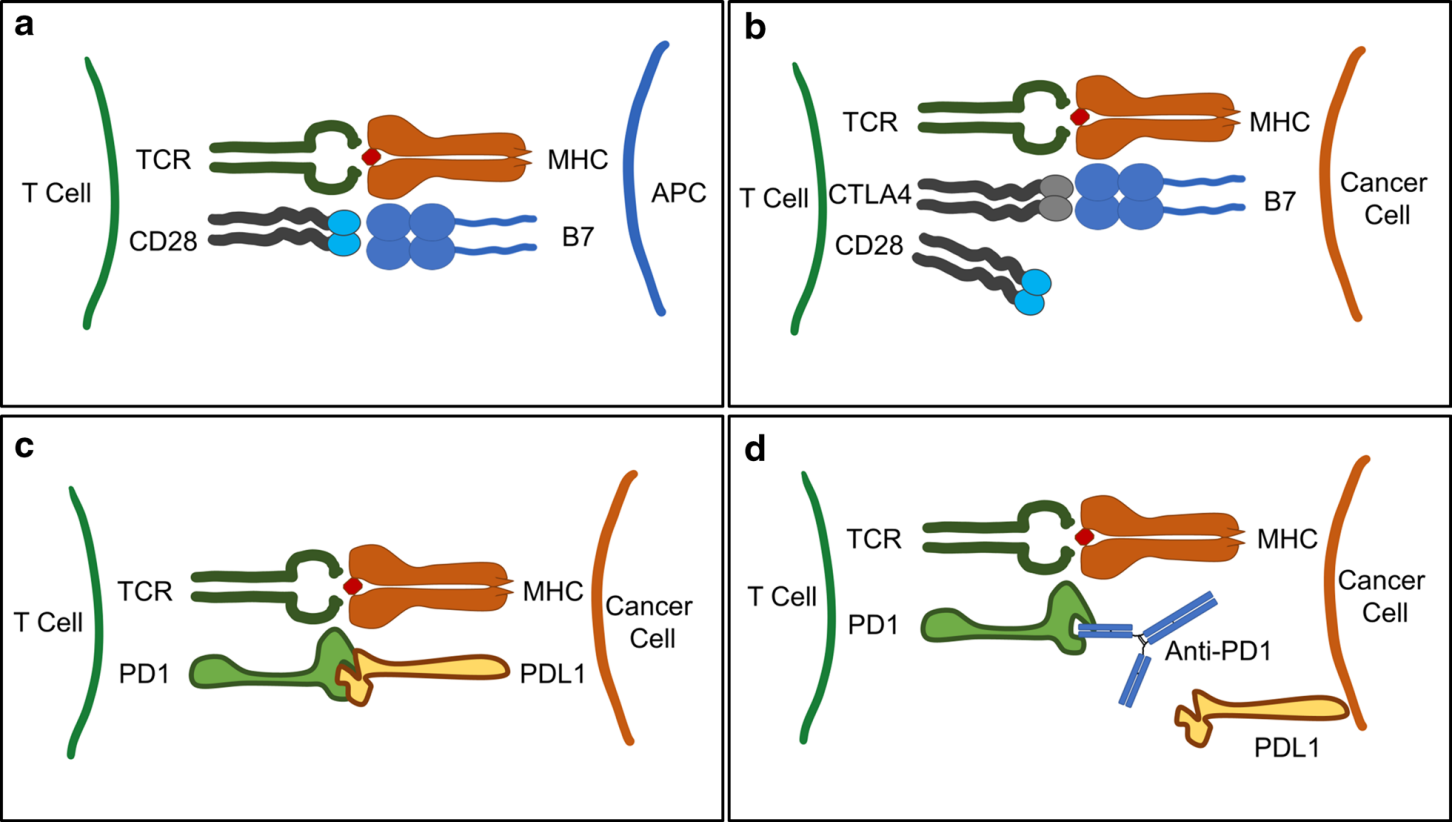
Schematic of T cell interactions. **a** Major histocompatibility complex (MHC) on antigen presenting cell (APC) binding to T cell receptor (TCR) along with the costimulatory B7-CD28 interaction. **b** CTLA-4 competitively inhibits the binding of B7 to CD28 and results in dampened T cell activation and proliferation. **c** PD-1 on a T cell binding to PD-L1 expressed on a cancer cell to decrease T cell activation. **d** Anti-PD-1 antibody binding to PD-1 and eliminating the negative effect of the PD-1/PD-L1 axis on T cell function

**Fig. 2:**
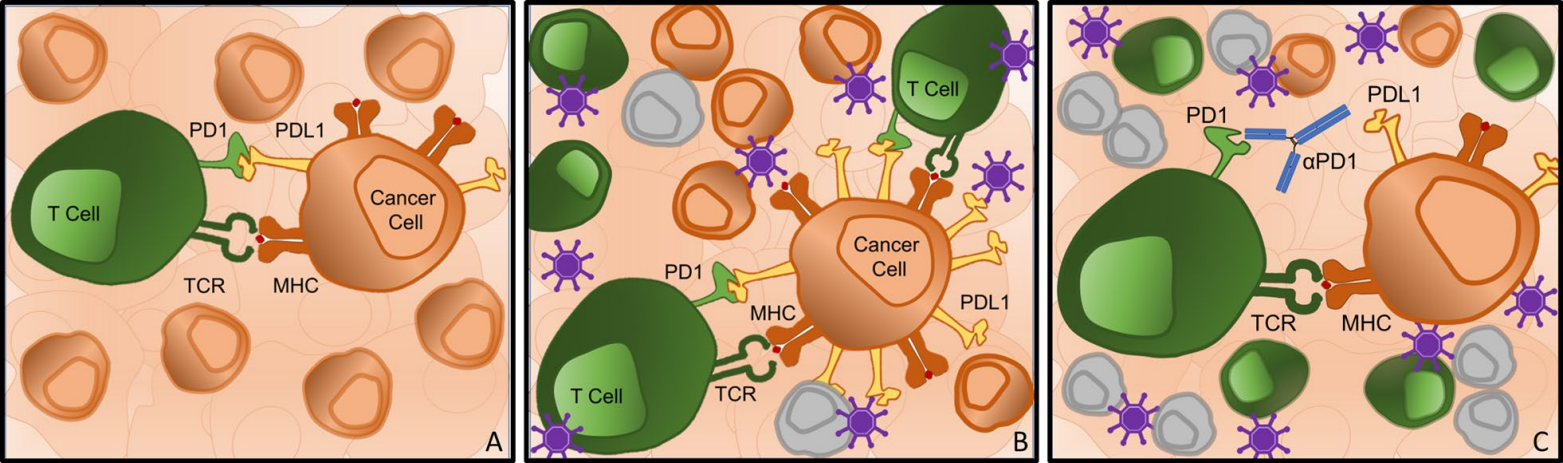
Combining oncolytic vectors and checkpoint inhibitors. **A** Illustration of the PD-1/PD-L1 axis between a T cell and cancer cell, which suppresses T cell activation. **B** Oncolytic viruses have the ability to directly lyse and kill cancer cells (grey cells), but also can exert a change in the local tumor microenvironment by increasing immune cell activation and PD-L1 expression on cancer cells. **C** Following priming by an oncolytic virus infection and transition to a ‘hot’ tumor microenvironment, checkpoint inhibitors (anti-PD-1 antibody) are more efficacious at decreasing T cell suppression

The activation of T cells is a multi-step process which begins with the major histocompatibility complex (MHC) on antigen presenting cells (APC) displaying antigens for recognition by T cell receptors. To achieve T cell activation, there must be a costimulatory signal in the form of B7 molecules on the APC surface binding to CD28 molecules on the T cell surface (Fig. 1a) [35]. CTLA-4 is a member of the immunoglobulin superfamily and is an inhibitory molecule expressed on the surface of activated T cells [36]. It competitively inhibits the binding of B7 to CD28 and effectively diminishes the degree of T cell activation and proliferation (Fig. 1b) [5]. Ipilimumab is one example of a monoclonal antibody that inhibits CTLA-4 and thereby increases T cell activation. PD-1 is a member of the immunoglobulin superfamily present on a variety of immune cells including activated T cells, B cells, NK cells, and antigen presenting cells [37]. Cytokines resulting from infection or tumor formation can induce the release of programmed death receptor ligand 1 (PD-L1), which negatively affects the function of T cells and B cells (Figs. 1c, 2A) [6]. Pembrolizumab is an example of an anti-PD-1 antibody which modulates the PD-1/PD-L1 axis to decrease the negative regulation on lymphocyte activation (Fig. 1d) [6, 13].

The TME is a complex milieu of inflammatory and immune cells that creates an environment which facilitates tumor growth [38]. As discussed previously, immunologically ‘cold’ tumors have a paucity of local infiltrating immune cells, while ‘hot’ tumors have a plethora of circulating immune cells [39]. This is important as immunologically ‘hot’ tumors are known to be more responsive to therapy with checkpoint inhibitors [40]. Therapeutics that can induce a migration of immune cells into the TME can be combined with checkpoint inhibitors to achieve an enhanced effect, and oncolytic viruses have thus far shown much promise in this regard (Fig. 2B, C) [41, 42].

## Oncolytic viruses in combination with checkpoint inhibitors

### Herpesviruses

Defining characteristics of the Herpesviridae family include a linear, double-stranded DNA genome, an icosahedral capsid, and a glycoprotein envelope [43]. These are large viruses that can be up to 200 nm in size with a genome that is approximately 150 kb [16]. Importantly, while viral replication occurs in the host cell nucleus, it does not cause insertional mutagenesis as it does not insert into the host genome [16]. There are eight members of this family that commonly infect humans, but Human herpesvirus 1 (a member of the alpha subfamily and more commonly known as herpes simplex virus type 1 [HSV-1]) has been the most studied and tested as a backbone for oncolytic vectors [44].

#### Talimogene laherparepvec (T-Vec)

The first FDA-approved oncolytic vector for use in the United States was talimogene laherparepvec (T-Vec). It has a HSV-1 backbone that is modified with deletions in ICP34.5 to augment the tumor selective replication of the virus [45]. T-Vec was also modified with deletions in the ICP47 gene to decrease neurovirulence and inclusion of the human GM-CSF transgene to augment the immune response via improved antigen presentation and T-cell priming [46]. Phase I studies of T-Vec demonstrated it to be a well-tolerated agent. For HSV-seronegative patients, the maximum tolerated single, intratumoral dose was 10^7^ plaque forming unit (pfu)/mL as this titer caused extensive local reactions of inflammation and erythema at the injection site [47]. Notably, HSV-seropositive patients had much less of a local cutaneous reaction. For a multi-injection cohort, seronegative patients were pre-treated with 10^6^ pfu/mL to seroconvert and then treated with two doses of either 10^7^ or 10^8^ pfu/mL with minimal cutaneous reactions [47]. Additionally, there were no obvious differences in clinical response between HSV-seropositive and HSV-seronegative patients in this study [47]. In the phase III OPTiM trial, injection of intralesional T-Vec demonstrated a statistically significant improvement in durable overall response rate when compared to GM-CSF alone (16.2% vs 2.1%, p < 0.001) in patients with unresectable stage IIIB or IV melanoma [48]. Additionally, 15% of measureable visceral (uninjected) lesions reduced in size by 50% or more following treatment with T-Vec [48]. Importantly, intralesional injection with T-Vec into metastatic melanoma lesions alters the immune cell makeup of the tumor microenvironment as demonstrated by a decrease in multiple suppressor cell populations including CD4^+^ Tregs, CD8^+^ T suppressor cells, and myeloid derived suppressor cells (MDSC) [49]. Finally, T-Vec-induced local and systemic Melanoma Antigen Recognized by T cells (MART)-1-specific CD8^+^ effector cells, which suggests the establishment of anti-tumor immunity [49].

Given the distinct mechanisms of action of T-Vec and the checkpoint inhibitor ipilimumab (anti-CTLA-4), researchers have postulated that their combined effect on the immune microenvironment and T cell modulation may be greater than either of the monotherapies. In a phase Ib study, T-Vec was combined with ipilimumab in patients with untreated stage IIIB or IV melanoma. The objective response rate was 50% and 44% of patients enrolled had a durable response that lasted at least 6 months [50]. There were no dose-limiting toxicities and overall adverse event rates were comparable to that of ipilimumab monotherapy. Interestingly, the authors observed that patients who demonstrated better disease control had increased levels of activated CD8^+^ T cells (flow cytometry analysis of whole blood samples) when compared to those patients who had disease progression following T-Vec monotherapy [50]. This difference did decrease following ipilimumab, and consequently the authors suggest that T-Vec might stimulate a different subset of T cells that could generate a more specific anti-tumor response when compared to those stimulated by ipilimumab [50]. In the subsequent phase II trial, 39% of patients in the combination arm (T-Vec + ipilimumab) demonstrated an objective response while only 18% of patients in the ipilimumab arm demonstrated a response (p = 0.002) [51]. Additionally, distant un-injected sites demonstrated abscopal responses as decreases in visceral lesions were demonstrated in 52% of patients in the combination arm but only in 23% of patients in the ipilimumab arm [51]. The combination of T-Vec with an anti-CTLA-4 antibody has demonstrated a greater efficacy while still maintaining tolerability and has the potential to become a standard therapy for patients with advanced melanoma.

T-Vec is also being tested in combination with an anti-PD1 antibody (pembrolizumab) for the treatment of melanoma patients. The ongoing Masterkey-265 trial was designed as a phase Ib/III trial, and thus far results have been promising (NCT 02263508). In the phase Ib study, there were no dose limiting toxicities and the objective response rate was 62% with a complete response rate of 33% [41]. The authors demonstrated increased levels of PD-L1 expression, increased amounts of circulating CD8^+^ and CD4^+^ T cells, and increased inflammation at tumor sites distant from the injected lesions (even in patients with low levels of tumor infiltrating lymphocytes [TIL]) [41]. These analyses were performed prior to anti-PD1 antibody delivery, which suggests that the oncolytic virus can change the immune cell makeup surrounding the tumor which is then more conducive to combination therapy with a checkpoint inhibitor. A phase III study is ongoing and the results are highly anticipated.

#### HF 10

HF 10 is another oncolytic virus in the HSV family. It differs from T-Vec in that it is a spontaneously mutated virus without any insertions of foreign genes. Natural deletions and insertions resulted in an overexpression of UL53 and UL54 as well as a loss of expression of UL43, UL49.5, UL55, and UL 56 [52]. This has translated into high innate tumor tropism, a high degree of viral replication, and potent antitumor efficacy across a variety of malignancies [52]. It has been used in a phase II clinical trial with patients who have Stage IIIB/C or IV unresectable melanoma in combination with ipilimumab (NCT 02272855). There were no disease limiting toxicities, and the best overall response rate was 41%, while the disease stability rate was 68% [53].

### Adenoviruses

The Adenoviridae family consists of non-enveloped viruses with double-stranded DNA genomes that are surrounded by an icosahedral capsid [54]. These viruses range from 70 to 90 nm in size and possess a genome of approximately 35 kb that is relatively easy to modify and lends itself well to the insertion of transgenes [16]. From the 57 known serotypes of adenovirus (divided into categories A–G based upon their agglutination properties and oncogenic potential in rodent models), serotype 5 from group C has been one of the most commonly used backbones in oncolytic viruses [55].

#### Tasadenoturev (DNX-2401)

This is a replication competent oncolytic adenovirus with enhancements to confer increased infectivity as well as tumor selectivity [56]. The selective replication of the vector results from a 24 base pair deletion in the E1A region of the adenoviral genome, which allows the virus to replicate in cancer cells that lack a functional Rb pathway, but not in normal cells [57]. The vector was tested in a phase I trial for thirty-seven patients with recurrent malignant glioma. One group (n = 25, group A) underwent intratumoral injections to evaluate dosing and response across different viral titers, while the other group (n = 12, group B) underwent intratumoral injection via implanted catheter and subsequent surgical resection. Tumor size reductions were documented in 72% of patients in Group A with a median overall survival time of 9.5 months [58]. Immunohistochemical analysis of resected specimens demonstrated decreases in the expression of TIM-3, but none of the other checkpoint proteins including PD-1 or PD-L1 [58]. T-cell exhaustion is one of the ways that tumor cells can create a locally immunosuppressed environment, and it is known that inhibitory receptors (e.g. TIM-3, PD-1) can provide some regulation of these exhausted T cells [59]. The authors suggest that since viral inoculation with DNX-2401 may partially overcome some aspects of T-cell exhaustion and have subsequently used this as a rationale to investigate the virus in combination with anti-PD-1 antibodies [58]. The CAPTIVE trial is an ongoing phase II study employing the virus and pembrolizumab in patients who have had glioblastoma that has progressed after initial therapy (NCT 02798406).

#### ONCOS-102 (Ad 5/3 Δ24 GM CSF)

ONCOS-102 is a serotype 5 adenovirus with multiple modifications including a chimeric 5/3 fiber-knob region to augment infectivity, a 24 base pair deletion in the E1a region conferring selective replication in Rb-pathway deficient cells, and expression of GM-CSF to boost the immune cell infiltrate at the site of viral inoculation [60]. This virus has been extensively tested in a pre-clinical setting and has already progressed to phase I trials. In a study of 12 patients with treatment refractory solid tumors (including mesothelioma, sarcoma, ovarian, colorectal, liver and lung cancers), there were no grade 4/5 adverse events following intratumoral injections [61]. Inoculation  with the vector resulted in a profound immune cell infiltration to the tumor. When compared to pre-treatment biopsies, ONCOS-102 resulted in a 5.9 times increase in the expression of CD3 (a T cell marker) and a four-fold increase in CD8^+^ cells in the tumor on post-treatment biopsies [61]. Additionally, two patients (one with mesothelioma, the other with ovarian cancer) developed systemic anti-tumor immunity as demonstrated through the comparison of pre and post treatment peripheral blood mononuclear cells (PBMC) in blood samples to determine the specificity of CD8^+^ T-cells for cancer-testis (CT) antigens [61]. Here, the mesothelioma patient demonstrated a profound induction of MAGE-A3-specific CD8^+^ T cells, and the ovarian cancer patient demonstrated CD8^+^ T cells that were specific for NY-ESO-1 [62, 63]. Furthermore, two of the patients with mesothelioma demonstrated increased levels of PD-L1 expression in the tumors following treatment with ONCOS-102 [61]. These observations serve as the basis for potential combination therapies with checkpoint inhibitors, and there is an ongoing clinical trial utilizing ONCOS-102 with pembrolizumab in patients with advanced melanoma who have progressed after PD1 blockade (NCT 03003676).

#### Other adenoviruses

Thus far, we have only discussed approaches whereby the drug of interest (checkpoint inhibitor) is given separately from the oncolytic vector. This has been described as a ‘trans approach’ while a ‘cis approach’ would consist of a gene encoding the product of interest (human monoclonal antibody for anti-PD1 or anti-CTLA-4) being inserted into the viral genome [64].

The Hemminki group has reported an oncolytic adenovirus that included a transgene expressing an antibody specific for CTLA-4 [65]. The vector (Ad 5/3 Δ24a CTLA4) was shown to produce a high level of the human monoclonal antibody against CTLA4, which was effective at inducing T cell activity [65]. Du et al. also reported construction of an oncolytic adenovirus with an anti-CTLA4 antibody inserted into the E3 region (SKL002), which was efficacious in both in vitro and in vivo models [66]. This vector was under the control of the E2F-1 promoter, which resulted in selective replication in cancer cells deficient in the Rb pathway. Notably, the vector demonstrated strong in vivo effects in subcutaneous mouse models for lung cancer and melanoma.

### Vaccinia viruses

Vaccinia viruses are members of the Poxviridae family and have large (~ 190 kb) double-stranded DNA genomes that are suitable for transgene insertion [67]. These viruses replicate in the host cytoplasm and so the risk of insertional mutagenesis is all but eliminated [16]. Due to its role in smallpox vaccination programs, the potential for immune system stimulation and the safety profile of this vector system is well documented [68]. These viruses do not have a specific cell-surface receptor required for entry into the host, which contributes to the natural tropism for a variety of cancer cells and makes it an attractive backbone for oncolytic virotherapy [69].

#### Pexa-Vec

Pexa-Vec (pexastimogene devacirepvec, JX-594) is an oncolytic vaccinia virus that expresses the human GM-CSF and beta-galactosidase transgenes [70]. In addition, it has an inactivated thymidine kinase gene that provides for selective replication that is dependent upon high levels of thymine production, which is common to many cancer cells [71]. In addition to its oncolytic and immunostimulatory properties, Pexa-Vec is also known to target vascular cells within the tumor. It has been demonstrated in laboratory and in human studies that this virus is capable of targeting and infecting tumor associated endothelial cells, which ultimately results in vascular disruption and oncolysis [72]. The vector has been tested in multiple clinical trials and has been shown to be well tolerated with antitumor activity across a range of solid malignancies [73–76]. In a recent abstract presented at the 2018 ASCO meeting, Anthoney et al. presented data in patients with metastatic liver tumors who were given a single dose of intravenous (IV) Pexa-Vec and then underwent surgical resection [77]. They demonstrated a robust activation of natural killer cells, antigen presenting cells, and CD4/CD8^+^ T cells. The study will go on to explore combinations of the viral vector with nivolumab for the treatment of liver tumors (NCT 03071094). Furthermore, Pexa-Vec is currently being studied in conjunction with immune checkpoint inhibitors in two additional ongoing clinical trials for colorectal cancer and other advanced solid tumors (NCT 03206073, NCT 02977156).

### Reoviruses

Reoviruses are members of the Reoviridae family and are typically 75–85 nm in diameter [16]. They are non-enveloped viruses with icosahedral capsids and double-stranded RNA genomes [78]. Reoviruses replicate in the cytoplasm and produce viral RNAs that activate the PKR (protein kinase R) pathway [16]. Interestingly, in Ras-transformed cells, the PKR pathway is inhibited which results in the release of translational inhibition and serves to augment the replication and oncolysis of reoviruses [79]. Given the number of cancers with Ras mutations, the natural tropism of reovirus for these tumors makes it a versatile backbone for oncolytic vector design [80].

#### Reolysin (Pelareorep)

Reolysin is a live, replication competent reovirus that is an isolate of the human Reovirus Type 3 strain [81] and mediates oncolysis through modulation of the Ras signaling pathway [79].

Recent preclinical data supports the use of the combination of reovirus and anti-PD-1 antibodies. In subcutaneous melanoma tumors established in immunocompetent mice, the combination of intratumoral reovirus and intravenous anti-PD-1 antibody (delivered 7 days after first viral dose) resulted in statistically significant prolonged survival compared to either monotherapy treatment [82]. The authors also showed that PD-1 blockade enhanced the CD8^+^ T cell Th1 antitumor response (primed by the reovirus injection) and also augmented NK-cell recognition of reovirus-infected cells [82]. Similar findings were obtained with intravenous reovirus injection in patients with intracranial glioblastomas. In a phase Ib trial, patients who were undergoing debulking neurosurgery underwent a reovirus infusion prior to resection. When compared to control cases, these patients demonstrated a marked increase in tumor-infiltrating cytotoxic T cells (CD8^+^) on immunohistochemical staining of the resected specimens [42]. A clinical trial (NCT 02620423) investigating the use of pelareorep in combination with pembrolizumab and chemotherapy in patients with relapsed metastatic pancreatic adenocarcinoma is ongoing. A recent abstract reported the preliminary data for 11 patients treated with pembrolizumab, pelareorep, and gemcitabine. The authors note manageable toxicities and 3 out of 5 patients who were eligible for efficacy evaluation demonstrated a partial response or stable disease [83].

### Coxsackie viruses

These viruses are members of the Picornaviridae family and are non-enveloped with a single-stranded RNA genome [84]. They are small viruses that are approximately 30 nm in size and have an icosahedral capsid structure [16]. There are two subgroups of Coxsackieviruses which are categorized based on their effects in murine models and consist of twenty-three unique serotypes in Group A and six serotypes in Group B [16, 85].

#### CVA 21 (Cavatak)

This coxsackie vector is the twenty-first serotype from Group A and has not been modified with any deletions or transgene insertions [85]. Its binding to cancer cells is mediated through intercellular adhesion molecule 1 (ICAM-1) and decay-accelerating factor (DAF) [86]. Since melanoma cells are known to overexpress ICAM-1, metastatic melanoma was one of the first targets for this oncolytic vector [87, 88]. Additionally, other serotypes of coxsackieviruses including CVA 13, CVA15, and CVA 18 are currently being explored for their potential as oncolytic vectors [89].

Additional pre-clinical studies have demonstrated an immune component to the anti-tumor response represented by increases in IL-8 and gamma-interferon in melanoma patients [90]. In addition, the vector was quite effective when combined with anti-PD-1 or anti-CTLA-4 antibodies [91]. Results of an extension study to the CALM clinical trial (NCT 01636882) showed increased immune cell infiltrates and expression of checkpoint molecules in patients receiving intratumoral injections of the CVA 21 virus [92]. These observations  and the strong cytocidal effects have led to multiple clinical trials, many of which have combined CVA 21 with immune checkpoint inhibitors [93–95]. For example, in the CAPRA clinical trial (single arm, multi-institutional, phase Ib, NCT 02565992) where patients received multiple intratumoral injections of CVA21 as well as multiple doses of pembrolizumab, there was an objective response rate of 73% [95].

## Summary and conclusions

The last decade has ushered in a new age of cancer care due to the mainstream adoption of immunotherapies. Checkpoint inhibitors have revolutionized the treatment of patients with melanoma and other malignancies. Similarly, with the FDA approval of T-Vec, oncolytic virotherapy gained a major victory. As researchers have learned more about the mechanism of action of viral oncolysis, it has become clear that the immune component is equally important (if not more so) than direct lysis. Consequently, the combinations of viral vectors with agents that influence the tumor immune microenvironment and help to augment T cell responses have incredible potential. Multiple vector systems are currently being tested in clinical trials in combination with anti-PD-1 or anti-CTLA-4 antibodies, and thus far the results have been encouraging. Additionally, many research groups are exploring similar treatment schemes with other vectors systems in preclinical settings, some of which include measles virus [96, 97], vesicular stomatitis virus (VSV) [98, 99], newcastle disease virus (NDV) [100, 101], semlicki forest virus (SFV) [102], and parvovirus [103]. With time, it is expected that additional oncolytic vectors with be tested with checkpoint inhibitors in clinical trials.

As these types of combination therapies progress in development, important points will need to be addressed. Will it be more beneficial to have the vector express a transgene which encodes for an anti-PD-1/CTLA-4 antibody or will concurrent IV delivery of such an antibody be best? If the latter is to be pursued, what will be the optimal timing of the antibody delivery?

Oncolytic vectors have the ability to lyse target cells as part of the replication cycle, but they also have powerful immunomodulatory effects. They have been shown to induce both innate and adaptive tumor responses as well as prime cancer cells for treatments with additional agents. To this end, multiple mechanisms (innate, adaptive, and acquired) of resistance to immunotherapy have been identified and oncolytic vectors are suited to be part of the solution to these hurdles in treatment [39, 104]. Multiple groups have demonstrated that local oncolytic virus injection can modulate tumor-specific CD8^+^ T-cell responses to make distant tumors increasingly susceptible to immune checkpoint inhibitor therapy [100, 105]. Results of ongoing clinical trials in patients who have progressed after immune checkpoint inhibition (e.g. NCT 03003676) will shed additional light on oncolytic virotherapy’s role in helping to overcome resistance to immunotherapy. By harnessing the potential of the combination of viral vectors and checkpoint inhibitors, great strides can be made in further developing treatment regimens employing these novel therapeutics to improve patient outcomes.

## Abbreviations

TNBC: triple negative breast cancer
RCC: renal cell cancer
NSCLC: non-small cell lung cancer
HNSCC: head and neck squamous cell carcinoma
HL: Hodgkin lymphoma (classic)
PMBCL: primary mediastinal B cell lymphoma
HCC: hepatocellular carcinoma
MSI-H: microsatellite instability high
dMMR: mismatch repair gene deficient
CTLA-4: cytotoxic T lymphocyte-associated antigen-4
PD-1: programmed death receptor 1
PD-L1: programmed death receptor ligand 1
GM-CSF: granulocyte macrophage colony stimulating factor
ICD: immunogenic cell death
TME: tumor-associated microenvironment
DAMP: damage associated molecular pattern
PAMP: pathogen-associated molecular pattern
PRR: pattern recognition receptor
APC: antigen presenting cell
DC: dendritic cell
NK: natural killer
PBMC: peripheral blood mononuclear cell
MDSC: myeloid derived suppressor cell
ICAM: intercellular adhesion molecule
DAF: decay accelerating factor
PSA: prostate-specific antigen
Cox2: cyclooxygenase-2
TERT: human telomerase reverse transcriptase
NIS: sodium iodide symporter
VP: viral particle
pfu: plaque forming unit
TCID: tissue culture infective dose
IT: intratumoral
IV: intravenous
SBRT: stereotactic body radiotherapy
CI: checkpoint inhibitor
HSV: herpes simplex virus
VSV: vesicular stomatitis virus
NDV: newcastle disease virus
SFV: semlicki forest virus
T-Vec: talimogene laherparepvec
Pexa-Vec: pexastimogene devacirepvec
Reolysin: pelareorep
